# Vaccinations and Chronic Diseases: Knowledge, Attitudes, and Self-Reported Adherence among Patients in Italy

**DOI:** 10.3390/vaccines8040560

**Published:** 2020-09-25

**Authors:** Francesco Napolitano, Giorgia Della Polla, Maria Simona Capano, Michela Augimeri, Italo Francesco Angelillo

**Affiliations:** Department of Experimental Medicine, University of Campania “Luigi Vanvitelli”, 80138 Naples, Italy; francesco.napolitano2@unicampania.it (F.N.); giorgia.dellapolla@unicampania.it (G.D.P.); simo86_6@libero.it (M.S.C.); michela.augimeri@unicampania.it (M.A.)

**Keywords:** chronic patients, coverage, Italy, recommended vaccinations, vaccine-preventable diseases

## Abstract

The aims of this cross-sectional survey were to evaluate the knowledge, attitudes, and self-reported adherence to recommended vaccinations among a random sample of patients with chronic conditions presenting for a medical visit in out-patient clinics in Italy. Patients who were healthcare workers (HCWs), those with diabetes, those who had received information by Internet, physicians, and friends/relatives, and those who needed more information were more likely to know that the influenza vaccine is recommended for patients with chronic diseases. More than half (58.2%) and 8.9% self-reported to have received at least one recommended vaccination and more than one, respectively. Patients who believed that vaccine-preventable diseases (VPDs) were dangerous for them, those who had received information by physicians, and those who needed information were more likely to have received at least one recommended vaccination. This behavior was less likely in married patients, those who were worried about the side effects of the vaccines, and those who suffered from renal failure. The results highlight the need to implement effective vaccination programs in order to decrease the complication of VPDs in at-risk population.

## 1. Introduction

Vaccine-preventable diseases (VPDs) may be the cause of severe complications for patients at-risk due to age, immune system impairment, and health status [1], and they consume a large consumption of resources [2,3]. Indeed, immunization programs have been implemented worldwide to promote vaccination of patients with chronic conditions such as cardiovascular, metabolic, and neoplastic diseases that could lead to an increase in disability and mortality, associated with VPDs [4,5,6].

In Italy, in the latest Immunization Plan, vaccinations against *haemophilus influenzae* type b, hepatitis A, hepatitis B, herpes zoster, influenza, measles/mumps/rubella, meningococcal disease, pneumococcal disease, and varicella are recommended and provided free of charge for patients with chronic conditions [7] by general practitioners or vaccination public centers. However, the immunization rates are inadequate [8,9,10,11], despite the extraordinary impact of the vaccinations on the reduction or the elimination of severe and deadly infectious diseases. Vaccinations coverage has been influenced by various determinants such as a progressive decrease in the awareness of the dangerousness of VPDs, hesitancy, access to healthcare services, lack of confidence in prevention measures, and fear about the side effects of vaccines [9,12,13,14,15,16]. It is well-known that the effectiveness of immunization programs depends on both the vaccine advice and recommendations by physicians, and the knowledge and beliefs about the usefulness of vaccines and the complications of VPDs among at-risk groups. Therefore, the evaluation of the level of knowledge of chronic patients and understanding of barriers to vaccination are useful in implementing preventive strategies in order to improve trust in vaccines and coverage.

Several studies have assessed the knowledge and the acceptability of recommended vaccinations in patients with chronic conditions, focusing mainly on single vaccines [17,18,19,20,21,22], but no such studies have been reported in Italy to the best of our knowledge, and the reasons for the low coverage have not been well investigated [10,11,21]. Therefore, the aims of this survey were to evaluate the knowledge of and attitudes and adherence to recommended vaccinations among patients with chronic diseases in Italy.

## 2. Materials and Methods 

### 2.1. Survey Design

A cross-sectional survey was carried out between June and December 2019 in the metropolitan area of Naples, Italy. A two-stage sampling strategy was used to select the participants. First, from the list of the public hospitals in the area, four were selected by simple random sampling. Then, the research team randomly approached potential participants aged 18 years and above when they came in for consultation, as outpatients at the selected hospitals in clinics for endocrine and metabolic disorders, cardiology, pulmonary, oncology, dialysis, and gastroenterology. Patients with psychiatric problems or who were unable to respond to interview were excluded. The sample size was calculated assuming an expected proportion of vaccinated patients with chronic diseases for each recommended vaccine of 30% [23,24,25], a confidence interval (CI) of 95%, and an error rate of 5%. A 20% non-response rate was calculated and added, which increased the estimated sample size to 405.

The directors of the selected hospitals received an invitation letter to request permission to perform the investigation with a description of objectives and methodology, and the assurance of anonymity and confidentiality regarding data collection. After approval, participants during their waiting time in the clinics were approached by two trained research assistants in data collection through face-to-face interview and informed of the purposes of the survey, its voluntary nature, and that they could withdraw at any stage. Participants gave their signed informed consent prior to data collection, and no financial compensation or gift was given. 

A pilot survey was conducted with 25 patients, not included in the final sample, to evaluate the clarity and comprehensiveness of the instrument. The protocol and the questionnaire were approved by the Ethics Committee of the Teaching Hospital of the University of Campania “Luigi Vanvitelli” (protocol number 217/2019).

### 2.2. Survey Instrument

The questionnaire incorporated five sections. The first section asked about patients’ socio-demographic and clinical characteristics (gender, age, marital status, education level, employment status, number of children, type of chronic conditions, number of medications, self-rated health status). The self-rated health status was evaluated through a 10-point Likert scale ranging from a value of 1 (very poor) to 10 (excellent). The second section asked about their knowledge of recommended vaccinations for patients with chronic conditions. Response options included “yes”, “no”, or “do not know”. The third section evaluated attitudes to VPDs and the relevant vaccinations (concern about the danger of VPDs, usefulness of the recommended vaccinations, concern about the danger of vaccinations relating to their health status, concern about vaccines’ side effects). The patients’ attitudes were measured on a 10-point Likert scale ranging from 1 to 10 with higher values corresponding to a stronger attitude. The fourth section asked about behaviors regarding recommended vaccinations for patients with chronic diseases (having talked with the physician, having received, reasons for not having received, willingness to receive). Response options included “yes” or “no” and a selection from a list of choices. The last section asked about the source of information on the recommended vaccinations and the need for more information. Response options included “yes” or “no” and a selection from a list of choices. 

### 2.3. Statistical Analysis

Descriptive statistics have been used to summarize all main socio-demographic and clinical characteristics of the patients. Then, to identify determinants associated with the different outcomes of interest, bivariate and multivariate logistic regression analyses were conducted. Independent variables included in the multivariate logistic regression models were those associated with bivariate analysis, using chi-square test for the categorical variables, and Student’s *t*-test for continuous, with *p*-value less or equal to 0.25 according to Hosmer and Lemeshow’s model building strategy [26]. Multivariate logistic regression analysis was used to identify determinants of the following four outcomes of interest: participants’ knowledge that influenza vaccine is recommended for those with chronic diseases (no = 0; yes = 1) (Model 1); participants’ attitude towards the usefulness of the administration of recommended vaccinations to patients with specific chronic conditions (1–5 = 0; 6–10 = 1) (Model 2); participants’ positive attitude toward willingness to receive recommended vaccinations (no = 0; yes = 1) (Model 3); and participants’ having received at least one recommended vaccination (no = 0; yes = 1) (Model 4). For the purposes of analysis, the outcome variable of Model 2, originally consisting of multiple categories, was categorized into two levels, with respondents grouped according to whether they reported a positive attitude, with a value greater than five, versus all others. The following potential determinants were considered in all multivariate logistic regression models: age (continuous), gender (male = 0; female = 1), marital status (married = 1; other = 0), number of children (continuous), education level (none or primary/middle/high schools = 0; college degree or higher = 1), working as healthcare worker (HCW) (no = 0; yes = 1), underlying chronic disease (cardiovascular or pulmonary = 1; cancer = 2; liver = 3; kidney = 4; diabetes = 5), sources of information about the recommended vaccinations (none = 0; internet = 1; mass media = 2; physicians = 3; friends/relatives = 4), and need for additional information about the recommended vaccinations (no = 0; yes = 1). Variables (knowledge that patients with chronic diseases are at greater risk of complications from VPDs (no = 0; yes = 1), knowledge that the recommended vaccinations are safe for patients with chronic diseases as well as for healthy subjects (no = 0; yes = 1), knowledge that vaccinations do not cause an exacerbation of their chronic disease (no = 0; yes = 1), and believing that VPDs are dangerous for them (continuous), were included in Models 2 to 4. Moreover, variables (believing that the administration of recommended vaccinations is useful for patients with chronic diseases (no = 0; yes = 1), fear of the vaccines’ side effects (continuous), and considering the vaccinations administered as dangerous (continuous)) were included in Models 3 and 4.

A stepwise procedure was used to build the final models and the significance levels for exclusion and inclusion of variables were *p*-values of 0.4 and 0.2, respectively. The results of the logistic regression analyses were reported as odds ratios (ORs) and 95% confidence intervals (CIs). All inferential tests were two-tailed and determinants were considered significantly associated with the outcome for *p*-values equal to or less than 0.05. Statistical analyses were performed using the software Stata 15 [27].

## 3. Results

### 3.1. Socio-Demographic and Anamnestic Characteristics 

Of the 450 approached patients, 414 participated, a 92% response rate. The principal characteristics of the survey population are described in Table 1. More than half were females, the average age was 60.1 years, 77.3% were married, 11.8% had a college degree or higher education level, 56.8% were employed and 6.5% were HCWs, 6.5% had more than one chronic disease, and the average self-rated health status was 6, on a scale ranging from 1 to 10.

### 3.2. Knowledge of Recommended Vaccinations

Among all respondents, only 8.2% knew all the recommended vaccinations for adults with chronic disease, 26.6% knew one, and 71% declared that they did not know them at all. Overall, 22.7% of patients correctly acknowledged that the influenza vaccine is recommended for adults with chronic diseases, whereas only 11.5% knew of the pneumococcal vaccination. When asked about the recommended vaccinations for patients with specific chronic conditions, only 12.4% with cardiovascular diseases or diabetes knew that herpes zoster vaccine is recommended for them. Moreover, 16.1% and 14.8% of those with chronic hepatitis correctly indicated that they should undergo the hepatitis B and hepatitis A vaccines, respectively. An even lower level of knowledge was found for other recommended vaccinations, since 8.8% of patients with chronic hepatitis, renal failure, and diabetes knew that measles/mumps/rubella vaccination is recommended for these chronic conditions, and only 8.2% of those with cancers, renal failure, chronic hepatitis, and diabetes correctly answered that they are recommended to receive vaccination against meningococcal disease. Moreover, 7.7% of participants with renal failure, diabetes, or chronic hepatitis correctly knew the varicella vaccination recommendation.

The results of the multivariate logistic regression model showed that patients who were HCWs (OR = 11.22; 95% CI = 4.31–29.24), those with diabetes (OR = 2.94; 95% CI = 1.59–5.43), compared to those with cardiovascular and pulmonary diseases, those who had received information on recommended vaccinations by internet (OR = 4.92; 95% CI = 1.53–15.81), physicians (OR = 3.88; 95% CI = 1.88–8.01), and friends/relatives (OR = 3.55; 95% CI = 1.31–9.58) compared to those who had not received information, and those who needed more information (OR = 2.12; 95% CI = 1.24–3.62) were more likely to know that the influenza vaccine is recommended for patients with chronic diseases (Model 1 in Table 2).

Approximately one-third (36%) of respondents knew that the recommended vaccinations were safe for patients with chronic diseases as well as for healthy subjects, 43.2% indicated that these patients are at greater risk of complications from VPDs, and only 31.6% were aware that vaccinations do not cause an exacerbation of their disease.

### 3.3. Attitudes towards VPDs and Recommended Vaccinations

A large majority of participants (84.5%) felt that VPDs are dangerous for them, with a mean value of 7.7, on a scale ranging from 1 to 10, while 39.6% considered the recommended vaccinations as being dangerous with an average value of 5.3, and 55.1% were worried about the side effects of the vaccines with a mean value of 5.9, on a scale ranging from 1 to 10. Almost two-thirds (64.7%) believed that the administration of the recommended vaccinations is useful for them with a mean value of 7 on a scale ranging from 1 to 10. The multivariate logistic regression analysis indicated that patients who believed that the VPDs are dangerous for them (OR = 1.3; 95% CI = 1.15–1.47), those who knew that recommended vaccinations are safe for them as well as for healthy subjects (OR = 3.69; 95% CI = 1.96–6.96), those who had received information on vaccinations by mass media (OR = 5.5; 95% CI = 2.36–12.85) compared to those who had not received information, and those who needed more information (OR = 7.89; 95% CI = 3.89–16.01) were more likely to have a positive attitude toward the usefulness of the recommended vaccinations. On the other hand, male patients (OR = 0.57; 95% CI = 0.34–0.97) and those who believed that vaccinations could cause an exacerbation of their disease (OR = 0.37; 95% CI = 0.19–0.72) were less likely to have this positive attitude (Model 2 in Table 2). 

Among unvaccinated patients, 42.7% were willing to receive the recommended vaccinations. The results of the multivariate logistic regression analysis showed that this attitude was significantly higher in those with a college degree or higher education level (OR = 9.17; 95% CI = 1.67–50.31), those who knew that patients with chronic diseases are at greater risk of complications from VPDs (OR = 2.74; 95% CI = 1.06–7.11), and those who had received information on vaccinations by physicians (OR = 9.35; 95% CI = 1.93–45.17) compared to those who had not received information. Moreover, patients who believed that vaccinations could cause an exacerbation of their disease (OR = 0.23; 95% CI = 0.07–0.75) and those who were worried about the side effects of the vaccines (OR = 0.73; 95% CI = 0.58–0.92) were not willing to receive the recommended vaccinations (Model 3 in Table 2). The most common reported reasons for a positive attitude were fear of the infectious diseases (50.7%), considering themselves at risk (37.3%), and having been advised by physicians (12%). 

### 3.4. Self-Reported Behaviors about the Recommended Vaccinations

Table 3 shows the self-reported vaccinations adherence in the survey population. More than half (58.2%) had received at least one recommended vaccination and 8.9% more than one. The highest rate was for seasonal influenza in the previous year (48.5%) ranging from 27.3% for patients with cancers to 60.5% with chronic hepatitis. Only 9.4% of patients were vaccinated against pneumococcal disease in the last three years and the highest coverage was for those with diabetes (23.9%) and the lowest (10.1%) for those with cardiovascular and pulmonary diseases. One in ten patients (11.9%) with chronic hepatitis or renal failure had been immunized against hepatitis B and 8.7% against hepatitis A. The self-reported coverage for varicella and measles/mumps/rubella vaccines among susceptible patients with renal failure, diabetes, or chronic hepatitis was 3.9% and 1.9%, respectively. Moreover, none of the eligible participants had been vaccinated against herpes zoster and meningococcal disease. The most common reported reasons for not having received vaccinations were concerns about side effects (36.8%), considering themselves not at risk (31%), objection to the administration of vaccines (16.6%), and lack of recommendation from physicians (14.8%). Among patients who had received the recommended vaccinations, 82% declared that the general practitioners recommended these, whereas only 3.6% indicated the specialist, and 14.4% had the vaccination without consulting a physician.

The results of the multivariate logistic regression model showed that patients who believed that the VPDs were dangerous for them (OR = 1.21; 95% CI = 1.07–1.36), those who had received information on recommended vaccinations by physician (OR = 2.22; 95% CI = 1.29–3.83) compared to those who had not received information, and those who needed more information (OR = 2.31; 95% CI = 1.32–4.06) were more likely to have received at least one recommended vaccination. Instead, married patients (OR = 0.44; 95% CI = 0.22–0.87), those who were worried about the vaccines’ side effects (OR = 0.65; 95% CI = 0.57–0.75), and those with renal failure (OR = 0.46; 95% CI = 0.26–0.88), compared to those with cardiovascular and pulmonary diseases, were less likely to have received at least one recommended vaccination (Model 4 in Table 2). 

### 3.5. Sources of Information

A large majority (96.4%) of participants had received information about vaccinations from a variety of sources, including physicians (54.6%), mass media (25.6%), friends/relatives (9.4%), and the internet (5.8%). Moreover, 36.7% needed additional information on vaccinations.

## 4. Discussion

These findings are particularly important given, to the best of our knowledge, the dearth of information regarding knowledge, attitudes, and behaviors on recommended vaccinations among patients with chronic diseases in Italy and the results indicate that there is a need for more health promotion and targeted education of patients.

Respondents did not have full knowledge about recommended vaccinations. Indeed, only one in four were aware about influenza, and even lower numbers about measles/mumps/rubella and pneumococcal vaccinations. Moreover, only one-third knew that the recommended vaccinations are safe for patients with chronic diseases as well as for healthy subjects and that they do not cause an exacerbation of their disease. These findings were in accordance with those on adulthood in the same geographical area, in which only 35.4% knew that vaccination against influenza is recommended for patients with chronic diseases [9]. A lower level was found in the US [28], where only 19.6% of adults aged ≥18 years were knowledgeable, whereas higher levels, although in different populations, were found in France [29] and Saudi Arabia [30] where 39% and 36.7%, respectively, knew that people with chronic diseases should receive a vaccine, and in Turkey where almost half of the hospitalized participants stated that pneumococcal vaccination was necessary [31]. The low level of knowledge is worrying because it could be a barrier to the demand for preventive measures that can improve the health status of patients with chronic diseases. Therefore, educational interventions are needed and physicians, the main source of information in this sample, have a central role in the dissemination of appropriate information on vaccination.

The participants had a positive attitude regarding the usefulness of the vaccinations and a large majority felt that VPDs were dangerous for them. These findings confirmed previous investigations in the same geographical area [9,32,33]. The multivariate logistic regression analysis showed that receiving information and considering vaccinations to be safe are stronger predictors of this positive attitude. The results pointed out the need for efforts of policy makers and HCWs to improve public awareness regarding the efficacy and safety of vaccinations and the danger of VPDs in patients with chronic conditions. Therefore, it is necessary to improve physicians’ training and communication and to strengthen the preventive measures in integrated care plans for patients with chronic diseases among HCWs (mainly physicians) and vaccination services. 

Despite the positive attitudes, an overall low adherence, far below the target of 75% established by the National Immunization Plan, to the recommended vaccinations was observed. Indeed, only almost half of participants had been immunized against influenza and a small proportion against pneumococcal disease. Patients with cancer showed the lowest coverage. Oncologists should pay more attention to recommending vaccinations, because neoplastic diseases and related treatments can increase the patient’s susceptibility to infectious diseases with higher risk of hospitalization and mortality than the general population [34,35]. Adherence to influenza vaccination in this survey was similar to that observed in Spain among subjects with high-risk chronic conditions [23]. In the US, 37%, 39%, and 41% of patients with diabetes were immunized against pneumococcal disease, hepatitis B, and influenza, respectively [26] and 35.7% and 29.1% of adults aged ≥18 years with chronic liver disease reported having received ≥1 dose and ≥3 doses of hepatitis B vaccine, respectively [24], whereas in this sample only one in five with chronic hepatitis had been immunized against hepatitis B. The low adherence underlined the need for a stronger engagement of specialists, for example in public outpatient clinics, in preventive activities by recording chronic non-immunized patients and actively offering the vaccines. Physicians have a key role in discussing and recommending vaccinations. In this regard, it is important to underline that just over half of the participants had received information from physicians and only 3.6% of those immunized declared that specialists made the recommendation. This is a very disappointing finding because chronic patients have frequent clinical consultations with specialists and a close trust relationship. Indeed, the findings of the multivariate logistic regression analysis showed that knowledge of and adherence to recommended vaccinations were higher in patients who had received information by physicians. Consistent with recent literature was the impact of physicians’ recommendation on adherence among different at-risk groups [9,36,37,38]. However, several barriers have been reported by physician to discussing vaccination with these patients, such as, for example, lack of time, lack of an effective reminder system, patients’ absence from regular visits, and clinical status [39,40,41]. This is of particular importance since general practitioners and specialists play a key role in the immunization landscape and should be influential sources given that patients may receive untrustworthy information. An encouraging result is that more than one-third of participants needed more information about vaccinations and this finding is in line with results among at-risk groups in the same geographical area [9,32,42].

Another interesting result was that those who were worried about side effects were less likely to have received a recommended vaccination. HCWs must be supported by healthcare services in promoting vaccinations and in increasing knowledge regarding the vaccines’ safety. Healthcare services should implement actions with evidence of effectiveness in increasing coverage such as easily access to healthcare facilities, active calling, patient reminder and recall systems, implementation of immunization information systems for the documentation of vaccinations received by patients, information leaflets, and active offer of vaccinations during hospitalization. Moreover, collaboration between public vaccination services, general practitioners and specialists is needed in order to monitor the effectiveness of the preventive strategies implemented and to collect data on coverage. HCWs may be supported in improving coverage and reducing the number of missed opportunities for vaccinations, providing information, tools and resources to help the education of patients, and integration of immunization with other services. Moreover, it should be taken into account that consent procedures based on opt-out approaches are likely to result in higher acceptance than using opt-in. Reaching optimal coverage against influenza and other VPDs is essential for this at-risk group, considering also the burden of the recent outbreaks of SARS-CoV-2 all over the world.

The results of the current survey should be interpreted with caution as there are some methodological limitations. First, the cross-sectional design does not enable the establishment of a causal relationship between outcomes of interest and predictors. Second, the enrolment of patients with chronic conditions from hospitals in a single geographic area in Italy may affect the generalizability of the findings. Third, this survey was reliant on self-reported data and some results should be cautiously interpreted, especially questions about vaccination status that were not validated by medical records, which are vulnerable to the effects of self-report bias. Fourth, data collection was made through interview and it is possible that there were social desirability and recall bias. Despite these limitations, findings from this survey contribute to characterizing some misconceptions and the low coverage regarding recommended vaccinations in patients with chronic diseases.

## 5. Conclusions

In conclusion, the results of this survey are significant in helping to highlight the gaps in the levels of knowledge and in the uptake of recommended vaccines among patients with chronic diseases and it is important to implement preventive strategies, such as a recall system by healthcare facilities, to take advantage of medical visits and hospitalization to actively offer vaccination, and to make an assessment of those who have not been immunized, in order to raise awareness of HCWs in order to recommend vaccinations.

## Figures and Tables

**Table 1:** Socio-demographic characteristics of the study population.

Characteristics	*N*	%
**Age, years**	60.1 ± 16.1 (18–90) *	
**Gender**		
Male	190	45.9
Female	224	54.1
**Marital status**		
Married	320	77.3
Other	94	22.7
**Employment status**		
Unemployed	179	43.2
Employed	208	50.3
Healthcare worker	27	6.5
**Educational level**		
None or primary school	110	26.6
Middle school	133	32.1
High school	122	29.5
College degree or higher	49	11.8
**Underlying chronic diseases**		
Kidney	110	26.6
Diabetes	99	23.9
Cardiovascular or pulmonary	81	19.6
Cancer	78	18.8
Liver	46	11.1
**Self-rated health status**	6.4 ± 2.1 (1–10) *	

* Mean ± Standard deviation (Range).

**Table 2:** Multivariate logistic regression models indicating associations between independent characteristics and the outcomes of interest.

Variable	OR	SE	95% CI	*p* Value
**Model 1.** Knowledge that influenza vaccine is recommended for patients with chronic diseases				
Log likelihood = −184.31, χ^2^ = 74.94 (9 df), *p* < 0.0001				
HCWs	11.22	5.48	4.31–29.24	<0.001
Sources of information				
None	1 *			
Physicians	3.88	1.43	1.88–8.01	<0.001
Internet	4.92	2.93	1.53–15.81	0.007
Friends/relatives	3.55	1.79	1.31–9.58	0.012
Need of additional information	2.12	0.58	1.24–3.62	0.006
Underlying chronic diseases				
Cardiovascular or pulmonary	1 *			
Diabetes	2.94	0.92	1.59–5.43	0.001
Cancer	1.67	0.59	0.84–3.32	0.144
Older	1.01	0.01	0.99–1.03	0.144
Males	0.76	0.2	0.49–1.27	0.294
**Model 2.** Positive attitude towards the usefulness of the recommended vaccinations				
Log likelihood = −181.2, χ^2^ = 175.02 (13 df), *p* < 0.0001				
Need of additional information	7.89	2.85	3.89–16.01	<0.001
Sources of information				
None	1 *			
Mass media	5.5	2.38	2.36–12.85	<0.001
Physicians	1.98	0.72	0.97–4.06	0.061
Internet	2.01	1.26	0.59–6.88	0.266
Belief that the VPDs are dangerous for them	1.3	0.08	1.15–1.47	<0.001
Knowledge that the recommended vaccinations are safe for patients with chronic diseases as well as for healthy subjects	3.69	1.19	1.96–6.96	<0.001
Belief that vaccinations can cause an exacerbation of their disease	0.37	0.13	0.19–0.72	0.004
Males	0.57	0.15	0.34–0.97	0.039
Underlying chronic diseases				
Cardiovascular or pulmonary	1 *			
Diabetes	0.65	0.21	0.34–1.22	0.180
Cancer	0.71	0.24	0.37–1.37	0.304
Knowledge that patients with chronic diseases are at greater risk of complications from VPDs	1.51	0.47	0.82–2.79	0.189
College degree or higher education level	1.56	0.75	0.6–4.02	0.359
**Model 3.** Positive attitude toward willingness to receive the recommended vaccinations				
Log likelihood = −80.1, χ^2^ = 54.06 (8 df), *p* < 0.0001				
Sources of information				
None	1 *			
Physicians	9.35	7.51	1.93–45.17	0.005
Mass Media	4.71	3.95	0.91–24.43	0.065
Friends/relatives	3.17	3.04	0.48–20.79	0.228
Being worried about the vaccines’ side effects	0.73	0.86	0.58–0.92	0.007
College degree or higher education level	9.17	7.96	1.67–50.31	0.011
Belief that vaccinations can cause an exacerbation of their disease	0.23	0.14	0.07–0.75	0.015
Knowledge that patients with chronic diseases are at greater risk of complications from VPDs	2.74	1.33	1.06–7.11	0.038
Need of additional information	2.36	1.19	0.87–6.35	0.09
**Model 4.** Having received at least one recommended vaccination				
Log likelihood = −212.82, χ^2^ = 123.89 (11 df), *p* < 0.0001				
Being worried about the side effects of the vaccines	0.65	1.05	0.57–0.75	<0.001
Belief that the VPDs are dangerous for them	1.21	0.07	1.07–1.36	0.003
Need of additional information	2.31	0.66	1.32–4.06	0.003
Sources of information				
None	1 *			
Physicians	2.22	0.62	1.29–3.83	0.004
Friends/relatives	1.72	0.73	0.75–3.93	0.201
Internet	1.97	1.11	0.65–5.95	0.229
Underlying chronic diseases				
Cardiovascular or pulmonary	1 *			
Kidney	0.46	0.13	0.26–0.88	0.007
Married	0.44	0.15	0.22–0.87	0.02
HCWs	0.48	0.26	0.17–1.37	0.172
Knowledge that the recommended vaccinations are safe for patients with chronic diseases as well as for healthy subjects	1.41	0.4	0.81–2.47	0.228
Males	0.8	0.2	0.49–1.3	0.378

* Reference category.

**Table 3:** Self-reported recommended vaccinations coverage for patients with specific chronic conditions.

Recommended Vaccination	Diabetes (*n* = 46)		Kidney (*n* = 78)		Chronic Hepatitis (*n* = 81)		Cardiovascular or Pulmonary (*n* = 99)		Cancer (*n* = 110)		Total of Eligible Patients Vaccinated	
	***n***	**%**	***n***	**%**	***n***	**%**	***n***	**%**	***n***	**%**	***n***	**%**
At least one vaccination	31	67.4	53	67.9	65	80.2	60	60.6	32	29.1	241	58.2
Seasonal influenza	23	50	46	59	49	60.5	53	53.5	30	27.3	201	48.5
Hepatitis B	-	-	3	3.8	16	19.8	-	-	-	-	19	11.9
Pneumococcal	11	23.9	7	9	6	7.4	10	10.1	5	4.5	39	9.4
Hepatitis A	-	-	-	-	7	8.7	-	-	-	-	7	8.7
Varicella	4	8.7	1	1.3	3	3.7	-	-	-	-	8	3.9
Measles/Mumps/Rubella	2	4.3	1	1.3	1	1.2	-	-	-	-	4	1.9
Meningococcal	2	0.5	-	-	-	-	-	-	-	-	2	0.5
Herpes zoster	-	-	-	-	-	-	-	-	-	-	-	-
*Haemophilus influenzae* type b	-	-	-	-	-	-	-	-	-	-	-	-

## References

[1] Doherty M., Schmidt-Ott R., Santos J.I., Stanberry L.R., Hofstetter A.M., Rosenthal S.L., Cunningham A.L. (2016). Vaccination of special populations: Protecting the vulnerable. Vaccine.

[2] Zhang D., Petigara T., Yang X. (2018). Clinical and economic burden of pneumococcal disease in US adults aged 19–64 years with chronic or immunocompromising diseases: An observational database study. BMC Infect. Dis..

[3] McLaughlin J.M.4, McGinnis J.J., Tan L., Mercatante A., Fortuna J. (2015). Estimated human and economic burden of four major adult vaccine-preventable diseases in the United States. J. Prim. Prev..

[4] World Health Organization Global Vaccine Action Plan 2011–2020. http://www.who.int/immunization/global_vaccine_action_plan/en/.

[5] Centers for Diseases Control and Prevention Vaccines and Immunizations for Specific Groups of People. https://www.cdc.gov/vaccines/spec-grps.html#conditions.

[6] European Centre for Disease Prevention and Control Guidance Priority Risk Groups for Influenza Vaccination. https://www.ecdc.europa.eu/sites/default/files/media/en/publications/Publications/0808_GUI_Priority_Risk_Groups_for_Influenza_Vaccination.pdf.

[7] Ministero della Salute Piano Nazionale Prevenzione Vaccinale 2017–2019. http://www.salute.gov.it/imgs/C_17_pubblicazioni_2571_allegato.pdf.

[8] Ministero Della Salute Dati Coperture Vaccinali. http://www.salute.gov.it/portale/influenza/dettaglioContenutiInfluenza.jsp?lingua=italiano&id=679&area=influenza&menu=vuoto.

[9] Napolitano F., Della Polla G., Angelillo I.F. (2019). Knowledge, attitudes, and behaviors of parents towards recommended adult vaccinations: An explanatory survey in the geographic area of Naples, Italy. Int. J. Environ. Res. Public Health.

[10] Gallone M.S., Infantino V., Ferorelli D., Stefanizzi P., De Nitto S., Tafuri S. (2018). Vaccination coverage in patients affected by chronic diseases: A 2014 cross-sectional study among subjects hospitalized at Bari Policlinico General Hospital. Am. J. Infect. Control.

[11] Gallone M.S., Martino S., Quarto M., Tafuri S., Bari Policlinico General Hospital (2017). Active offer of vaccinations during hospitalization improves coverage among splenectomized patients: An Italian experience. Am. J. Infect. Control.

[12] Bianco A., Mascaro V., Zucco R., Pavia M. (2019). Parent perspectives on childhood vaccination: How to deal with vaccine hesitancy and refusal?. Vaccine.

[13] Napolitano F., D’Alessandro A., Angelillo I.F. (2018). Investigating Italian parents’ vaccine hesitancy: A cross-sectional survey. Hum. Vaccines Immunother..

[14] Guthrie J.L., Fisman D., Gardy J.L. (2017). Self-rated health and reasons for non-vaccination against seasonal influenza in Canadian adults with asthma. PLoS ONE.

[15] Schmid P., Rauber D., Betsch C., Lidolt G., Denker M.L. (2017). Barriers of influenza vaccination intention and behavior—A systematic review of influenza vaccine hesitancy, 2005–2016. PLoS ONE.

[16] Chiatti C., Barbadoro P., Marigliano A., Ricciardi A., Di Stanislao F., Prospero E. (2011). Determinants of influenza vaccination among the adult and older Italian population with chronic obstructive pulmonary disease: A secondary analysis of the multipurpose ISTAT survey on health and health care use. Hum. Vaccines.

[17] Kurmi O.P., Davis K.J., Hubert Lam K.B., Guo Y., Vaucher J., Bennett D., Wang J., Bian Z., Du H., Li L. (2018). Patterns and management of chronic obstructive pulmonary disease in urban and rural China: A community-based survey of 25,000 adults across 10 regions. BMJ Open Respir. Res..

[18] Crouse Quinn S., Jamison A.M., Freimuth V.S., An J., Hancock G.R. (2017). Determinants of influenza vaccination among high-risk black and white adults. Vaccine.

[19] Hustache S., Moyroud L., Goirand L., Epaulard O. (2017). Hepatitis B vaccination status in an at-risk adult population: Long-term immunity but insufficient coverage. Eur. J. Clin. Microbiol. Infect Dis..

[20] Khan A., Dickens A.P., Adab P., Jordan R.E. (2017). Self-management behaviour and support among primary care COPD patients: Cross-sectional analysis of data from the Birmingham chronic obstructive pulmonary disease cohort. NPJ Prim. Care Respir. Med..

[21] Cheung K.W., Mak Y.W. (2016). Association between psychological flexibility and health beliefs in the uptake of influenza vaccination among people with chronic respiratory diseases in Hong Kong. Int. J. Environ. Res. Public Healt..

[22] Sampson R., Wong L., Macvicar R. (2011). Parental reasons for non-uptake of influenza vaccination in young at-risk groups: A qualitative study. Br. J. Gen. Pract..

[23] Martinez-Huedo M.A., Lopez-De-Andrés A., Mora-Zamorano E., Hernández-Barrera V., Jiménez-Trujillo I., Zamorano-Leon J.J., Jiménez-García R. (2020). Decreasing influenza vaccine coverage among adults with high-risk chronic diseases in Spain from 2014 to 2017. Hum. Vaccines Immunother..

[24] Yue X., Black C.L., O’Halloran A., Lu P., Williams W.W., Nelson N.P. (2018). Hepatitis A and Hepatitis B vaccination coverage among adults with chronic liver disease. Vaccine.

[25] Alcusky M.J., Pawasauskas J. (2015). Adherence to guidelines for hepatitis B, pneumococcal, and influenza vaccination in patients with diabetes. Clin. Diabetes.

[26] Hosmer D.W., Lemeshow S. (2000). Applied Logistic Regression.

[27] StataCorp (2017). Stata Statistical Software: Release 15.

[28] Lu P., Srivastav A., Santibanez T.A., Stringer M.C., Bostwick M., Dever J.A., Kurtz M., Williams W.W. (2017). Knowledge of influenza vaccination recommendation and early vaccination uptake during the 2015–16 season among adults aged ≥18 years—United States. Vaccine.

[29] Casalino E., Ghazali A., Bouzid D., Antoniol S., Pereira L., Kenway P., Choquet C., The Emergency Department Study Group on Respiratory Viruses (2018). Patient’s behaviors and missed opportunities for vaccination against seasonal epidemic influenza and evaluation of their impact on patient’s influenza vaccine uptake. PLoS ONE.

[30] Alqahtani A.S., Althobaity H.M., Al Aboud D., Abdel-Moneim A.S. (2017). Knowledge and attitudes of Saudi populations regarding seasonal influenza vaccination. J. Infect Public Health.

[31] Ahmad Hamidi A., Gelmez Taş B., Gündüz A., Nur Çelebi S., Esen E.S., Toprak D., Dökmetaş İ (2018). Immunization rates of pneumococcal, influenza and tetanus vaccines and knowledge and attitudes of adult patients who receive inpatient treatment at hospital: Point prevalence study. Hum. Vaccines Immunother..

[32] Bertoldo G., Pesce A., Pepe A., Pelullo C.P., Di Giuseppe G., Collaborative Working Group (2019). Seasonal influenza: Knowledge, attitude and vaccine uptake among adults with chronic conditions in Italy. PLoS ONE.

[33] Pelullo C.P., Di Giuseppe G. (2018). Vaccinations among Italian adolescents: Knowledge, attitude and behavior. Hum. Vaccines Immunother..

[34] Eliakim-Raz N., Vinograd I., Zalmanovici Trestioreanu A., Leibovici L., Paul M. (2013). Influenza vaccines in immunosuppressed adults with cancer. Cochrane Database Syst. Rev..

[35] Cooksley C.D., Avritscher E.B.C., Bekele B.N., Rolston K.V., Geraci J.M., Elting L.S. (2005). Epidemiology and outcomes of serious influenza-related infections in the cancer population. Cancer.

[36] Jiang Y., Zhang X., Lv Q., Qi J., Guo X., Wei Q., Liao Z., Lin Z., Gu J. (2019). Knowledge, attitude, and practice regarding infection and vaccination in patients with rheumatic diseases in China. Hum. Vaccines Immunother..

[37] Tsachouridou O., Georgiou A., Naoum S., Vasdeki D., Papagianni M., Kotoreni G., Forozidou E., Tsoukra P., Gogou C., Chatzidimitriou D. (2019). Factors associated with poor adherence to vaccination against hepatitis viruses, *streptococcus pneumoniae* and seasonal influenza in HIV-infected adults. Hum. Vaccines Immunother..

[38] Wu S., Su J., Yang P., Zhang H., Li H., Chu Y., Hua W., Li C., Tang Y., Wang Q. (2017). Factors associated with the uptake of seasonal influenza vaccination in older and younger adults: A large, population-based survey in Beijing, China. BMJ Open.

[39] Böhm S., Roöbl-Mathieu M., Scheele B., Wojcinski M., Wichmann O., Hellenbrand W. (2019). Influenza and pertussis vaccination during pregnancy—Attitudes, practices and barriers in gynaecological practices in Germany. BMC Health Serv. Res..

[40] McNamara M., Buck P.O., Yan S., Friedland L.R., Lerch K., Murphy A., Hoge C. (2019). Is patient insurance type related to physician recommendation, administration and referral for adult vaccination? A survey of US physicians. Hum. Vaccines Immunother..

[41] Srivastav A., Black C.L., Lutz C.S., Fiebelkorn A.P., Ball S.W., Devlin R., Pabst L.J., Williams W.W., Kim D.K. (2018). U.S. clinicians’ and pharmacists’ reported barriers to implementation of the Standards for Adult Immunization Practice. Vaccine.

[42] D’Alessandro A., Napolitano F., D’Ambrosio A., Angelillo I.F. (2018). Vaccination knowledge and acceptability among pregnant women in Italy. Hum. Vaccines Immunother..

